# Modelling of risk effect of mercury on nutrient transformation in lake sediments

**DOI:** 10.1080/13102818.2014.946316

**Published:** 2014-10-20

**Authors:** Stilyana Lincheva, Irina Schneider, Elmira Daskalova, Yana Topalova

**Affiliations:** ^a^ Faculty of Biology, Sofia University ‘St. Kliment Ohridski’, Sofia, Bulgaria

**Keywords:** mercury, modelling, sediments, hydrochemical, microbiological indicators

## Abstract

The influence of mercury on the transformation processes in the waters and the sediments in the reservoir of a small hydroelectric power plant (SHPP) Lakatnik was simulated in laboratory conditions. SHPP Lakatnik is the first (of nine similar) SHPPs put into exploitation in the middle part of the Iskar River (Bulgaria). In previous studies of the sediments in the reservoir of SHPP Lakatnik, our team found high concentrations of mercury, sometimes exceeding maximum concentration limit (MCL). In model bioreactors we studied the effect of mercury on the dynamics of the following hydrochemical and microbiological indicators: NH_4_^+^, NO_3_^−^, NO_2_^−^, PO_4_^3−^, chemical oxygen demand (COD), total organic carbon (TOC); aerobic and anaerobic heterotrophs (AH and AnH), *Pseudomonas* spp. (Ps.), *Acinetobacter* spp. (Ac.), sulphate-reducing bacteria (SR), denitrifying microorganisms (Dn). We looked for linear correlations among the studied indicators in order to find quick, mutual replaceability among them. The extent to which mercury affects the amount of key microbial groups and the rate of transformation of biogenic elements was calculated in percentages. The microorganism dynamics showed that AH, AnH and Ps. adapt more quickly and their number increased more in the presence of mercury, whereas SR, Dn and Ac. were inhibited to a greater extent by the presence of mercury. Mercury had a marked stimulating effect on Ps., which showed a 312% increase in number at the 192nd hour. The obtained results can be used when decisions have to be taken in risky situations related to increased concentrations of mercury in the sediments.

## Introduction

In the sediments of the Iskar River downstream from the city of Sofia, different organic and xenobiotic (heavy metals, oil products) pollutants have been accumulating for decades. These pollutants can go in the waters in risky situations.[1] Among the heavy metals accumulated in the sediments, mercury deserves special attention because of its unique characteristics such as high toxicity, instability and capability of bioaccumulation.

On a global scale, mercury is accumulated in the sediments by means of many physical, chemical, biological, geological and anthropogenic processes in the environment.[2–11] The direct (point) pollution with mercury is usually from abandoned mercury mines, gold-mining activities,[12–19] ore-mining and processes such as mercury recycling.[20–24]

A large amount of organic matter and xenobiotic pollutants (heavy metals, including mercury) are also contained in the sediment/water system sampled from the reservoir at the small hydroelectric power plant (SHPP) Lakatnik, which is the first (of nine similar) SHPPs in the middle part of the Iskar River (Bulgaria). Such sediment/water samples were used in the model experiment. The mercury concentration in the sediments exceeds the maximum concentration limit (MCL) according to data from monitoring report ‘Evaluation of the environmental effect of the ecosystem of the Iskar River and the technical condition of the facilities on the Middle Iskar cascade from 2011’.[25]

Until now, in this part of the river four SHPPs have been put into exploitation and the construction of five more is planned. An ecological problem in the functioning of the SHPP is the accumulation of sediments next to the wall of the small dams and the undergoing biodegradation of trivial and toxic pollutants. The conditions in the sediments are various – anoxic, anaerobic, aerobic in the upper layers and upon release of the sediments through the valves.[26] As a result of this release of sediments along the river course, physical, chemical or biological processes, the mercury buried in the sediments can be remobilized and released in the waters. This creates risky situations for the whole world of organisms and requires searching for solutions for their management. The modern approach to control of the management at such risky points is via bioalgorithms (graphic and mathematical), which allow adequate management of the processes undergoing *in situ*. As a contemporary form of control, bioalgorithms contribute to the consistent solution of critical problems regarding water purification.[1,27]

The aim of the completed studies is to evaluate the hazardous influence of mercury on the self-purification processes by means of analysis of its modulatory effect on key microbiological and hydrochemical parameters. This will be used to construct algorithms for management of the bioremediation potential of the sediments from the reservoir at the SHPP Lakatnik.

## Materials and methods

### Design of the experiment

**Figure 1.**
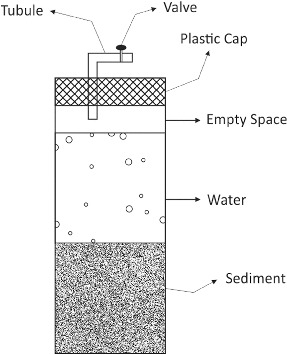
Experimental scheme of the model process.

To study the effect of mercury on key hydrochemical and microbiological parameters, two model systems were constructed. The basic part of each of the two includes a glass container with a tight-fitting cap (Figure 1), where 300 g of sediment are placed (taken from the uppermost layer of the bottom with standard Ekman bottom grab, with a length of 15 cm) and 300 mL of river water (taken from the water layer closest to the bottom – 5 m, taken with a bathometer). The intensive processes of biodegradation are carried out mainly on the border waters/sediments. The sediments and the waters were taken from the reservoir of the SHPP Lakatnik in March 2012. For each of the two model systems, seven of the above-mentioned glass containers were made. Each of them were maintained under the simulation conditions for a specified period of time and were analysed at 0, 24, 72, 120, 192, 264 and 336 h. The measurements were carried out at shorter time intervals (every 24 or 48 h) at the beginning of the experiment, after which proceeded at longer time intervals (every 72 h). The reason for this is the expected depletion of oxygen in the course of time and slowing down of the metabolic processes in the model systems.

The control (C) model system contained only 300 g of sediment and 300 mL of water from the reservoir of SHPP Lakatnik. In the other model system (denoted as +Нg), besides an equivalent amount of sediment and water there was also 0.6 mg/kg of Hg^2+^ added in the form of HgCl_2_ (0.8 mg/kg). Mercury was chosen for a model pollutant since preliminary studies of the sediments in the reservoir revealed it to be present at increased concentrations.

### Analytical methods

The concentration of organic matter in samples was measured as chemical oxygen demand (COD) and total organic carbon (TOC) according to the American Public Health Association (APHA).[28] The concentration of ammonium ions, nitrites, nitrates and phosphates was analysed by colorimetric methods, according to BNS-EN-ISO. An ultraviolet—visible spectrophotometer (Ultrospec 3000, Pharmacia Biotech Ltd.) was used.

The amount of key microbial groups (aerobic and anaerobic heterotrophs (AH and AnH), *Pseudomonas* spp. (Ps.), *Acinetobacter* spp. (Ac.), sulphate-reducing bacteria (SR) and denitrifying microorganisms (Dn)) was analysed according to the routine microbiological practice.[29] The results for the hydrochemical and microbiological indicators are average values from three repetitions, processed with the Microsoft Excel 2007 software. The standard deviations were determined on the basis of Guaranteed Analysis (0.95).

### Determination of the effect of mercury on transformation processes

In order to examine the transformation processes in the systems, we calculated the extent to which mercury affects the amount of key microbial groups (in percentages) and what its effect is on the rate of transformation of each of the studied biogenic elements. The amount of microorganisms, the nutrient concentrations and COD values were assumed to be 100% in C. The effect of mercury on the functional microbial structure and the rate of transformation of the biogenic elements (in percentages) compared to those in C, as well as correlations among different indicators were studied at the three key phases of the model experiment, also designated as follows: early phase (24–48 h), middle phase (120–192 h) and late phase (264–336 h).

The following formula for calculation of the transformation rate of nutrients was used:


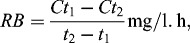


where *Ct*_1_ is the value of the parameter (NH_4_^+^, NO_3_^−^, NO_2_^−^, PO_4_^3−^, COD) on the 24th hour; *Ct*_2_ is the value of the parameters on the 48th hour for the early phase. The calculations for the middle and late phase were done by analogy. In this experimental work, only linear correlative dependencies are discussed (L) because the linear relation allows mutual replaceability of the correlating indicators.

## Results and discussion

### Dynamics of hydrochemical parameters

**Figure 2.**
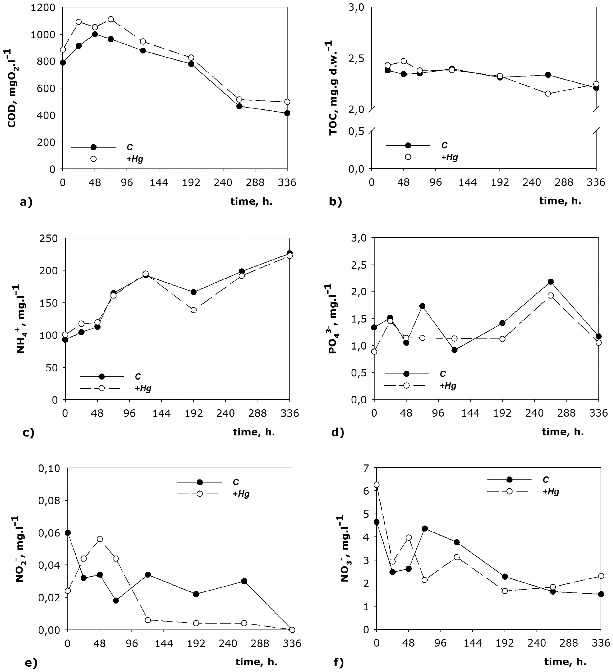
Dynamics of the nutrient transformation in the C and in the +Hg water/sediment system.

Figure 2 shows the dynamics of the nutrient transformation in both model systems (C and +Hg), where we found similar initial COD (Figure 2(a)). COD followed the same trend in both variants but the values in the +Hg system were higher. This is probably namely due to mercury, which forms different salts and increases COD. Mercury seemed to slow down the decrease of organic matter but the total decrease of COD from the beginning to the end of the process for the C system was 375 mg О_2_/L and for the +Hg system, 388 mg О_2_/L. Still in both simulation systems the residual organics as COD were measured to be 413 mg О_2_/L for the C one and 497 mg О_2_/L for the +Hg one, which is normal, considering the composition of the sediments in the two model systems and the lack of oxygen necessary for further decrease in the values of COD.

The data for TOC (Figure 2(b)) showed the same trend for a decrease in the organic matter from the beginning to the end of the model experiment. Similarly to COD, TOC was also higher in the +Hg system, but this was not so over the whole studied period, but only until the 72nd hour. Until the 264th hour the curves for the two variants had overlapping values, after which the TOC value for the +Hg system decreases and reached the lowest value for the model experiment (2.15 mg/g). At the 336th hour, TOC in the C system was again less than that in the +Hg one.

A large part of the organic matter in the dams is of protein nature. The phases of transformation of the proteins – hydrolysis and deamination – are related to accumulation of NH_4_^+^ in the sediments. The process of accumulation is derivative of two simultaneous processes: accumulation of ammonium ions and their depletion as a result of their inclusion in newly formed biomass. The ammonium ions in the hydrosphere are predominantly of biogenic origin and result from aerobic and anaerobic processes for degradation of nitrogen-containing organic substances (for example, ammonification), in which bacteria take part.[30]

The quantity of the ammonium ions (Figure 2(c)) increased from the beginning towards the end of the model experiment in both variants, which shows undergoing mineralization of the nitrogen compounds. In the +Hg system, a decrease in the ammonium ions concentration was observed only at the 192nd hour. This corresponds to the peaks in the growth of some groups of microorganisms exactly at this stage of the process, e.g. АH, АnH, Ps., which use ammonium ions for their metabolic needs.

Nitrates are highly water-soluble and are the most oxidized form of nitrogen. They are an indicator that the oxidation of the ammonium ions is completed. Figure 2(f) also shows that in both studied variant denitrification is underway. The concentration of the nitrates decreases with some fluctuations but as a whole there is a clear denitrification process during the whole studied period. The decrease in the concentration of nitrates in the course of the process is related to the depletion of oxygen used by the aerobic microorganisms as a main electronic acceptor. The decrease of the redox-potential allows facultative aerobic and anaerobic microorganisms to use final electron acceptors other than oxygen, e.g. nitrates.

**Figure 3.**
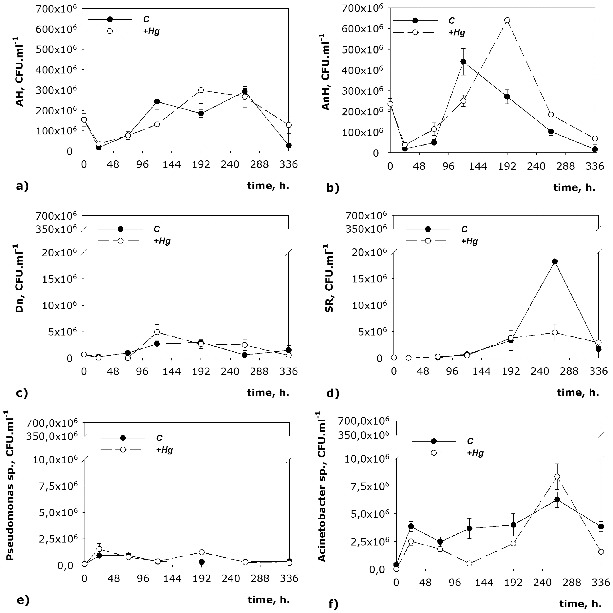
Dynamics of the quantity of key microbial groups in the C and in the +Hg water/sediment system.

The nitrites are a transitional product obtained from nitrification and denitrification. In the model systems studied by us the concentrations of nitrites (Figure 2(e)) had similar dynamics in both studied variants. At the beginning of the process their concentration in the +Hg was lower. After the 120th hour of the process their quantity became lower in the C system. At the end of the process the nitrite ions had similar concentrations in both variants. The nitrites are an unstable nitrogen form and during the whole experiment their concentration was low. Similar to the nitrates, the nitrites had higher concentrations at the beginning of the process than at the end, which is probably due to the presence of oxygen in the system. In the course of the experiment the decrease of the oxygen quantity led to using nitrites as a final electron acceptor. The increase in the phosphates concentration (Figure 2(d)) at the end of the experiment also confirms the presence of active mineralization processes but it is also possible that this increase could be related to the release of phosphates from the dead cells at the end of the process. The microorganism dynamics (Figure 3) showed that most of the microbial groups at the end of the model process significantly decreased in number.

### Dynamics of microbiological parameters

The quantity of the studied functional groups of microorganisms provides information about the rate and scale of the biodegradation water-purification processes (Figure 3). Considering the oxygen necessary for their growth, AH (Figure 3(a)) should be more at the beginning of the process and should decrease in its course. The fluctuations in the dynamics of AH in both systems, however, confirm the assumption that parts of the aerobic bacteria were facultative anoxic bacteria and facultative anaerobes. The effect of mercury on the quantity of АH and АnH in the course of the process became apparent after the 72nd hour (Figure 3(a) and 3(b)). This shows that at the beginning of the process, Hg does not significantly alter the number of the two types of microorganisms. After the 72nd hour of the process there was inhibiting influence of mercury on the amount of microorganisms from both types, which lasted until the 192nd hour, when these microorganisms were already more numerous than in the +Hg variant. At the end of the process (336th hour), the amount of AH and AnH in the +Hg system was again higher. This allows us to speculate that the inhibitory effect of mercury for these two types of microorganisms lasted only from the 72nd to the 120th hour of the process. Then the adaptation of microorganisms allowed their multiplication at an even faster rate than that observed in the C variant. It is thought that the dissolved organic matter limits the amount of the available non-organic mercury for the bacteria because part of the molecules of the dissolved organic matter (proteins, polysaccharides, lipids) are too big to pass through the cellular membrane of the bacteria.[31] Thus, the mercury bonded with organic matter does not have an inhibitory influence at the beginning of the process, which explains the almost identical number of microorganisms in both variants for AH, AnH, Ps. and SR until the 72nd hour.

The effect of mercury on the density of Ps. was most pronounced at the 192nd hour of the process (Figure 3(e)). In the remaining hours of the process, the quantities of the microorganisms in both variants were similar, indicating that Hg weakly inhibits the quantity of the bacteria from this genus. At the 192nd hour in the +Hg system, the number of these microorganisms increased significantly (312%) in comparison to the C system. This is probably a compensatory reaction to the inhibitory effect of mercury and an adaptive response of Ps. Another reason for the increase in the number of Ps. at the 192nd hour could be the strong decrease in the number of Ac. (Figure 3(f)). These two genera are main partners in the neutralization of toxic compounds and the decrease in the abundance of one of these genera could be a possible reason for the increase in the number of the other one.

The inhibitory effect of mercury on the number of Ac. was most noticeable at the beginning of the model process (Figure 3(f)). The increase in the number of Ac. observed at the 264th hour could probably be due to the reduced number of the representatives of Ps. at this point of the experiment. The compensatory reaction of this genus to the presence of mercury was shifted towards the 264th hour, where the number of microorganisms in the +Hg system was higher than that in the C one. The adaptation period for Ac. continued for a longer time, suggesting that the influence of mercury on this genus was stronger than on Ps. At the end of the model process, the quantities of Ac. and Ps. in the +Hg system decreased with 60% and 46%, respectively, in comparison to those in the C variant. This effect could be due to a combination of factors such as depletion of substrates and toxic influence of Hg.

An inhibitory effect of mercury on the amount of SR was observed only at the 264th hour of the process (Figure 3(d)). Being strict anaerobes, they had higher density in both variants after 192 h, which confirmed that at that particular point the conditions of the environment were predominantly anaerobic. At the 264th hour, the peak in the amount of SR was observed in both studied variants, the peak in the +Hg system being a lot smaller than the one in the C variant. The quantity of SR at the end of the experiment was greater in the +Hg system, which showed that the toxic influence of mercury had been quickly overcome. SR are obligate anaerobes and degrade the organic substrates in the bottom water layers and the sediment layers of dams. Besides organic pollutants, these bacteria can also bond ions of heavy metals in insoluble sulphides.[32] Hg (II) is a weak acid and easily forms complexes with weak bases as reduced sulphate compounds.[33] In this way insoluble compounds are formed, which are less toxic and settle down in the sediments. Adding reduced sulphur in soils containing mercury in order to achieve precipitation to HgS is a method used to stabilize soils contaminated with mercury. HgS is comparatively insoluble and less dangerous than the other forms. Precipitation of mercury from the activity of SR has been reported by King et al.[34]

The Dn were represented in small numbers at the beginning of the process, when the oxygen in the medium was not depleted and when nitrification processes prevailed. After the transition to anoxic/anaerobic conditions (at about 120 h), Dn increased in quantity (Figure 3(c)). These bacteria showed the greatest density at the 120th hour in the + Hg system.

### Effect of mercury on hydrochemical and microbiological parameters

**Figure 4.**
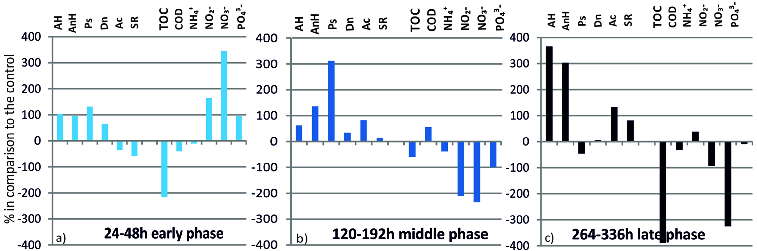
Effect of mercury on the functional microbial structure and the rate of nutrient transformation in the water/sediment system (in %) in comparison to the control.

In order to follow the transformation processes in the systems we expressed the extent to which mercury influences the quantity of key microbial groups and its effect on the nutrient transformation rate in percentages (Figure 4). The effect of mercury was calculated for the early (24–48 h), middle (120–192 h) and late phase (264–336 h). These phases are related, on the one hand, with the ecological specificity of the anaerobic process, and on the other hand, with the time of influence of Hg on the microbial structure and the rate of the transformation processes of the biogenic elements.

The modulating effect of Hg on the functional structure of the microbial community and on the rate of the transformation processes of the biogenic elements is extremely important for drawing out algorithms for managing this risk factor. During the early phase (Figure 4(a)) under the influence of Hg there was a pronounced increase in the quantity of AH, AnH, Dn and Ps. At the same time, the rate of transformation of organic matter, expressed as ТОС and COD, was inhibited. The rate of transformation of nitrites, nitrates and phosphates was activated in this early phase. All this is related to the preference of the microorganisms for other electronic acceptors in the conditions of oxygen depletion and decrease of the redox potential. The trend of the microbial supply with energy and its intensive consumption for increasing the quantity of certain microbial groups is also related to stimulation of the rate of transformation of phosphates, which are a basic factor for the formation and depletion of the adenosine triphosphate (ATP)-pool. During the middle phase of the process there was no inhibition of any of the studied groups of microorganisms and the great increase in the density of Ps. in comparison to the other studied microorganisms was apparent (Figure 4(b)). The great percentage increase of the representatives of Ps. under the influence of mercury can be explained again with the ability of these microorganisms to use alternative ways for energy supply as well as with the occupation of free microniches while suppressing the growth of the other microbial groups. During the middle phase of the process, the rate of transformation of the biogenic elements (expressed as ammonium ions, nitrites, nitrates, phosphates and TOC) was inhibited. Mercury did not have an inhibitory effect only on the values of COD. The suppression of the transformation of the biogenic elements under the influence of the heavy metal was observed during the late phase, too. During the last phase of the process, Hg had a strong inhibitory effect on the transformation of TOC (389%), nitrates (325%) and a weaker one on COD (32%), nitrites (92%) and phosphates (9%) (Figure 4(c)). During the late phase of the process the quantity of AH and AnH increased markedly (365% and 303) under the influence of Hg, whereas the effect on Ps. was negative (−46%). The quantity of Ac., as well as the amount of SR increased in the presence of mercury in this phase.

The relations between the effect of mercury and the percentage variation of microorganisms and the percentage variation of the rate of transformation of the biogenic elements in the sediment/water system during our experiment is shown in Table 1. Among some of the studied indicators we found linear correlations, which are interesting in terms of their practical application. These correlations can be applied when quick diagnostics is needed as well as a choice of a small number of very informative indicators about the management of the processes. Linear correlations were found between the effect of the mercury on the percentage variation of AnH, Dn, Ac and SR microorganisms during the three phases of the process. These linear correlations show that in the course of the experiment these groups of microorganisms were positively influenced by mercury as a modulator and increased in density from the early to the late phase of the process.

**Table 1.** Effect of mercury on the percentage variation of microorganisms and of the nutrient transformation rate during the early, middle and late phase in the sediment/water system.

MO	Effect of mercury on the quantity of key groups of microorganisms	Reliability
AH		*R*^2^ = 1
AnH		*R*^2^ = 0.891
Ps		*R*^2^ = 1
Dn		*R*^2^ = 0.99
Ac		*R*^2^ = 0.951
SR		*R*^2^ = 0.99
Rate of transformation	Effect of mercury on the rate of transformation of the biogenic elements	Reliability
ТОС		*R*^2^ = 1
СОD		*R*^2^ = 1
NH_4_^+^		*R*^2^ = 1
NO_2_^−^		*R*^2^ = 1
NO_3_^−^		*R*^2^ = 1
PO_4_^3−^		*R*^2^ = 1

Note: Shaded rows emphasize the linear correlation.

**Figure 5.**
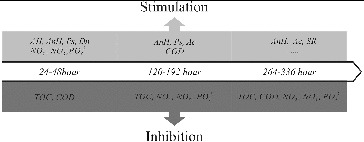
Bioalgorithm for management of the hazardous influence of mercury on the nutrient transformation in the sediment/water system.

The bioalgorithm for management of the hazardous influence of mercury on the nutrient transformation in the sediment/water system is presented in Figure 5. The addition of mercury in the sediment/water system inhibited the rate of transformation of organic matter during all three phases of the process. As the simulated process progressed, mercury showed an inhibitory effect on all of the studied hydrochemical indicators. The opposite was observed for the microbial groups. Examined in phases, the inhibitory influence of mercury was a lot weaker on the microbiological indicators, suggesting that in the presence of mercury the transformation processes of organic matter in the sediments are inhibited and the microbial groups compensate by increasing their quantity in order to overcome the toxic effect of mercury.

**Figure 6.**
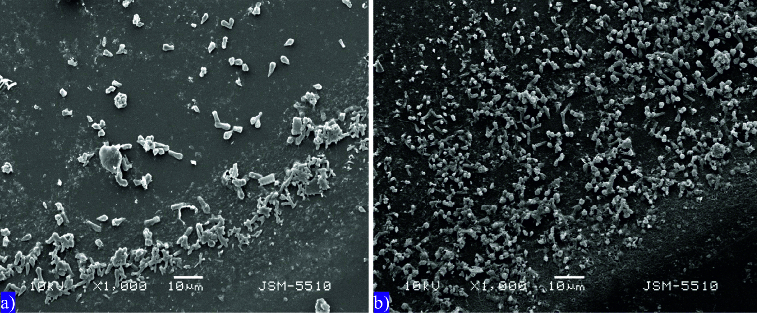
Scanning electron microscopy (1000 X) of sediment particle and aerophilic zone at 24th hour in the C (а) and the +Hg system (b).

The electron microscope visualization of the two studied systems has an important indicator value for the biological systems that carry out the water purification process.[35] In both photographs, a sediment particle and an aerophilic zone are shown (Figure 6). In both model systems the cells of the microbial community are smaller in size at the end of the zone. Deeper in the preparation the cells become larger. The cells in the C variant (Figure 6(a)) are less clearly visible in comparison to those in the +Hg system (Figure 6(b)). It is possible that the inhibitory effect of mercury on the transformation processes may have allowed better visualization. Probably the insoluble organic matter in the +Hg system was less, making the cells more visible. The thickness of the aerophilic zone is greater in Figure 6(b). Both microgrpahs show the edge of the drop, with all bacteria separated from the sediment matrix and having positive aerotaxis. In the +Hg system we found easier demobilization of the microbial cells from the sediment matrix and orientation towards the end of the drop. The increase of the aerophilic zone is an indicator for mobility and aerophilic properties. It is clearly seen that in this variant the microbial cells are more numerous, which suggests multiplication of the bacteria under the influence of mercury.

## Conclusions

The addition of mercury in the sediment/water system had an inhibitory effect on the transformation of organic matter. This shows that the presence of the toxic metal is related to permanent damage of the biodegradation potential. Mercury did not have a lasting inhibitory effect on the studied microbial groups. The stimulating effect of the toxic metal was greatest for *Pseudomonas* spp. (312%), which again confirms that upon loading with xenobiotic substrates, the microbial dominants are from this genus. Throughout the process, mercury resulted in a compensatory response, stimulating the growth of the anaerobic and aerobic facultative heterotrophs. This increase in the quantity of microorganisms can be a potential risk factor for the ecosystem because of the possibility of its secondary pollution.
